# Taylor’s Power Law rules the dynamics of allele frequencies during viral evolution in response to host changes

**DOI:** 10.1098/rsif.2025.0146

**Published:** 2025-07-16

**Authors:** João M. F. Silva, María J. Olmo-Uceda, Valerie J. Morley, Paul E. Turner, Santiago F. Elena

**Affiliations:** ^1^ Institute for Integrative Systems Biology (I2SysBio), CSIC-Universitat de València, Paterna, Valencia 46980, Spain; ^2^ Department of Ecology and Evolutionary Biology, Yale University, New Haven, CT 06511, USA; ^3^ QBio Institute, Yale University, New Haven, CT 06511, USA; ^4^ Microbiology Graduate Program, Yale School of Medicine, New Haven, CT 06511, USA; ^5^ Santa Fe Institute, Santa Fe, NM 87501, USA

**Keywords:** approximate Wright–Fisher diffusion model, effective population size, experimental evolution, Hurst’s exponent, selection coefficient, Sindbis virus, Taylor’s temporal fluctuation scaling, virus evolution

## Abstract

Sudden and gradual changes from permissive to resistant hosts affect viral fitness, virulence and rates of molecular evolution. We analysed the roles of stochasticity and selection in evolving populations of Sindbis virus under different rates of host replacement. First, approximate Markov models within the Wright–Fisher diffusion framework revealed a reduction in effective population size by approximately half under sudden host changes. These scenarios were also associated with fewer weak beneficial mutations. Second, genetic distance between populations at consecutive time points indicated that populations undergoing gradual host changes evolved steadily until the original host disappeared. Distances to the ancestral sequence in these cases exhibited occasional leapfrog phenomena, where the rise of certain haplotypes is not predictable based on their relatedness to previously dominant ones. In contrast, populations exposed to sudden changes exhibited less-stable compositions and diverged from the ancestral sequence at a consistent rate. Third, we observed that the distribution of allele frequencies followed Taylor’s Power Law. Both treatments exhibited high levels of allele aggregation and significant fluctuations, with neutral, beneficial and deleterious alleles distinguishable by their behaviour and position on Taylor’s plot. Finally, we found evidence that the host replacement regime influences the temporal distribution of mutations across the genome.

## 1. Introduction

Environmental heterogeneity strongly influences molecular adaptation dynamics. Pathogens adapt to new hosts by fixing mutations that enhance fitness in the novel host, even if these mutations are neutral or detrimental in the original host. Consequently, the availability of novel hosts in the environment affects both the rate and direction of molecular adaptation. For example, the adaptation dynamics of the Sindbis virus (SINV) to a less-permissive cell type in laboratory tissue culture are determined by the rate at which these novel host cells ‘invade’ the environment [1,2]. In a series of evolution experiments, Morley *et al.* [1] introduced a novel host cell type at varying rates, ranging from a gradual increase in the proportion of the novel cell type with each passage to an abrupt shift to an environment composed entirely of the novel host. Gradual changes were associated with virus populations achieving higher fitness in both novel and original hosts, as well as greater convergence among populations that fixed the same adaptive mutations [1]. Here, we reanalyse these results within a systems biology framework to uncover the interplay between selection and noise under different host replacement rates, and how this affects population composition during adaptation.

Allele frequencies typically fluctuate over time in both natural and experimental populations due to a variety of factors, including purely stochastic processes (e.g. genetic drift, fluctuating selection, migration or unpredictable ecological changes) and deterministic processes (e.g. directional selection). Indeed, temporal fluctuations are ubiquitous in physical systems, raising the question of whether they follow universal laws. Various power-law relationships have been found to be pervasive in physical and biological systems. One such relationship, known as Taylor’s Power Law—or the fluctuation scaling law—posits that the variance of a system’s *σ*^2^ elements scales as a power of the mean, *μ*: $\sigma^{2}=V\mu^{\beta }$ [3]. Originally described in ecology, this law naturally arises in many complex systems [4–9]. Its parameters capture both the amplitude of the noise level (*V*) and the degree of temporal aggregation (*β*) in the fluctuations observed within the system [5,6,8]. Notably, in the context of infectious diseases, Taylor’s Law has been applied to temporal data from the human microbiota, revealing that an individual’s negative health status is associated with increased noise and system instability [8]. Similarly, recent studies have found that during severe acute respiratory syndrome coronavirus 2 (SARS-CoV-2) infection, the transcripts dynamics in cells from human intestinal organoids, but not pulmonary cells, exhibit an increase–decrease–increase pattern in system noise and instability as infection progresses and the virus accumulates [9]. In the context of temporal variations in allele frequencies during virus adaptation to novel hosts, we propose that Taylor’s Power Law can be used to model how the variance of allele frequencies changes over time. In populations influenced solely by stochastic processes (i.e. exponential), it is expected that *β* = 2, meaning that variance scales quadratically with the mean allele frequency. In contrast, a *β* > 2 indicates non-random processes that amplify variability, such as migration or environmental heterogeneity, whereas a *β* < 2 suggests processes that constrain variability, such as stabilizing selection or density dependence (with *β* = 1 corresponding to a Poisson process).

Many natural processes exhibit long-term memory or persistent behaviour (autocorrelation), meaning that a high value is likely to be followed by another high value (and similarly for low values) [10–15]. This behaviour can be quantified using the Hurst exponent (denoted as *H* throughout this work), where: 0 < *H* < 0.5 indicates anti-persistent (negative autocorrelation) behaviour, *H* = 0.5 indicates a random walk, and 0.5 < *H* < 1 indicates persistent behaviour. Interestingly, many studied processes have an estimated *H* ≈ 0.7, a phenomenon known as the Hurst phenomenon [10]. Although Taylor’s Law and the Hurst phenomenon both describe variability and scaling behaviour, they focus on different aspects of the process and are indirectly related. Specifically, when Taylor’s Law is applied to the variance of fluctuations at different time scales, the simple relationship *β* = 2*H* must hold [11]. This connection arises because both laws describe the fractal or scaling properties of purely stochastic processes.

In this work, we estimate population genetic parameters—such as the selection coefficient per allele (*s*) and effective population size (*N_e_*)—and describe allele dynamics in experimentally evolving SINV populations under two different temporal schemes of host replacement. SINV populations that experienced a sudden replacement of a highly susceptible host with a less permissive one exhibited smaller *N_e_* values, indicating stronger bottlenecks and genetic drift. This observation prompted us to investigate how noise and selection affect the dynamics of virus molecular adaptation to novel hosts. First, we show that under gradual replacement of the highly susceptible host, the genetic composition of the viral populations changed steadily until the host was completely removed. In contrast, under the sudden treatment, the shift in population composition was more pronounced and less consistent. Second, we characterized Taylor’s Power Law for the temporal variation in allele frequency. This power model allowed us to examine in greater detail how selection and noise resulting from genetic drift influence the dynamics of molecular adaptation. Third, we investigate the persistent behaviour characteristic of the Hurst phenomenon in the distribution of mutations along SINV genomes, finding evidence that, in the sudden treatment, mutations become more randomly distributed earlier in time.

## 2. Methods

### 2.1. Description of the study system and data acquisition

**Figure 1.**
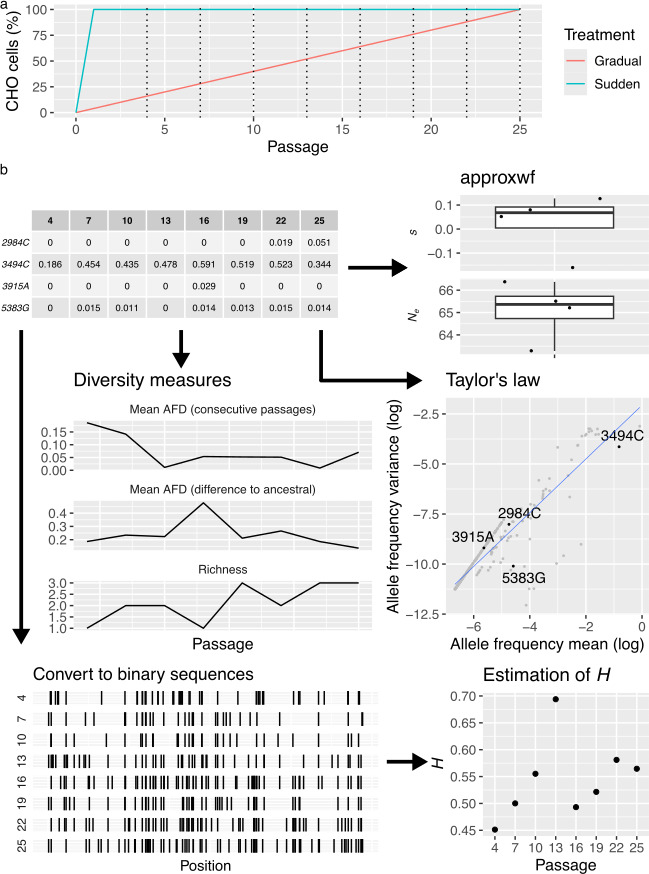
Schematic representations of the experiments and computational analyses flow. (a) Proportion of CHO cells at each passage for both treatments. Dotted lines represent the passages in which sequencing was performed. (b) Estimation of populational parameters, diversity measures and Taylor’s parameters directly from the alleles frequency table. Hurst’s exponent *H* was calculated from binary sequences where ones (represented by vertical lines) correspond to polymorphic sites.

We analysed data from an experimental evolution study that tracked the molecular adaptation of SINV (species *Alphavirus sindbis*, genus *Alphavirus*, family *Togaviridae*) typically cultured on a highly susceptible host, BHK-21 cells, while challenged to infect the less-susceptible host CHO cells, which were genetically modified (pgsD-677 ATCC CRL-2244) to be more resistant to SINV infection [1]. Briefly, SINV populations were evolved through 25 passages (approx. 100 virus generations) in monolayer cell cultures with a multiplicity of infection (MOI) of approximately 0.01 plaque-forming units (pfu) per cell at each passage. Two different treatments from the original study [1,2] were chosen for an in-depth analysis to investigate the link between population parameters and system dynamics of molecular adaptation to a novel host. Figure 1a shows a schematic representation of the chosen treatments. The initial stock was prepared by expression of an infectious clone in BHK-21 cells for 24 h [16]. In the gradual treatment, the proportion of CHO cells in the cell cultures increased at each passage, reaching 100% at the last passage. In contrast, in the sudden treatment, cell cultures were entirely composed of CHO cells from the first passage. For each treatment, nine populations were evolved and sequenced at passages 4, 7, 10, 13, 16, 19, 22 and 25. Following RNA extraction, two technical replicates were prepared for each sample from the reverse transcription step onward. Sample preparation is fully explained in [2].

The final allele frequency tables for each sample were obtained from [2]. Briefly, reads were trimmed with cutadapt version 1.8.3 [17], aligned to the consensus sequence of the original stock with BWA version 0.7.10 [18] and variant calling was conducted with QUASR version 7.01 [19] and VarScan version 2.3.9 [20]. To minimize technical errors, variants that appeared in only one technical replicate were assumed to be errors and were excluded. For variants detected in both technical replicates with a frequency of at least 1%, the mean of the two replicate frequencies was included in the final variant table.

### 2.2. Wright–Fisher approximate Bayesian computation estimates of selection coefficients and effective population sizes

The selection coefficient (*s*) and effective population size (*N_e_*) were estimated for each allele using the approxwf software [21] (figure 1b), which uses a discrete approximation of the Wright–Fisher diffusion model and Bayesian inference to estimate population parameters. The implementation assumes a log-uniform (1, 5) prior distribution on *N_e_* and a normal (0, 0.05) prior distribution on *s* [21]. The algorithm runs a Markov chain Monte Carlo (MCMC) using 51 states for 25 000 interactions, discarding the first 2000 interactions as burn-in. The mean values of *s* and *N_e_* from their posterior distributions were used in downstream analyses.

### 2.3. Genetic diversity within evolving viral populations

The alleles frequency table was used to calculate genetic distance and diversity between passages (figure 1b). To measure the genetic distance between two viral populations from consecutive time points, the allele frequency difference (AFD) [22] was calculated at each polymorphic site and averaged by the richness of the sample with custom R scripts, where n represents the total number of different alleles observed at the polymorphism and the *f_i_* terms are the proportion of allele i in the two viral populations,


$$AFD=\frac{1}{2}\sum_{i=1}^{n}\left|\left(f_{i1}-f_{i2}\right)\right|.$$

As a second measure of within-sample genetic diversity, we used allele richness, defined as the number of unique alleles per locus, adjusted for sample size.

### 2.4. Taylor’s Fluctuations Power Law

Mean and variance of allele frequencies (*p*) across time ($\langle p\rangle$ and $\sigma_{p}^{2}$, respectively) were computed and used to estimate Taylor’s parameters *V* and *β* by linear regressions in the log–log space: $log\sigma_{p}^{2}=logV+\beta log\langle p\rangle$ [3] (figure 1b). Given that the proportion of zeros had a significant influence on the fits, Taylor’s parameters were estimated independently for alleles grouped by their number of non-zero occurrences. Multivariate analysis of variance (MANOVA) of Taylor’s parameters were performed with parameters estimated from at least five observations. The magnitude of effects was evaluated using the $\eta_{P}^{2}$ statistic. Conventionally, 0.01 < $\eta_{P}^{2}$ ≤ 0.14 are considered as medium effects and $\eta_{P}^{2}$ > 0.14 as large ones. Three random walk matrices were constructed based on the cumulative sum of random variables drawn from a normal distribution with a mean of zero and standard deviations of 0.2, 0.05 and 0.01, respectively. For each matrix, 1000 random walks with eight time points were generated, and values below 0.01 were set to zero and those above 0.99 were set to one. After eliminating walks with only zeros, 792, 757 and 587 random walks were present in the matrices with standard deviation 0.2, 0.05 and 0.01, respectively.

### 2.5. Long-range dependence of mutation sites

To characterize the long-range dependence behaviour of mutations along the SINV genome, rescaled-range analyses were performed on the placement of mutations on each time point with the R package pracma version 2.4.2 [23]. For this analysis, binary sequences of length 11 703 (which is the genome length) with ones in the position of mutations were used to estimate the empirical Hurst’s exponent (*H*) (figure 1b). The minimum window size was set to 2000 to avoid windows with only zeros. Indels were excluded from this analysis.

### 2.6. Statistical analyses

All the statistical analyses indicated above were performed with R version 4.4.0 under RStudio version 2024.04.2+764.

## 3. Results and discussion

### 3.1. A sudden host transition is associated with stronger genetic drift

**Figure 2.**
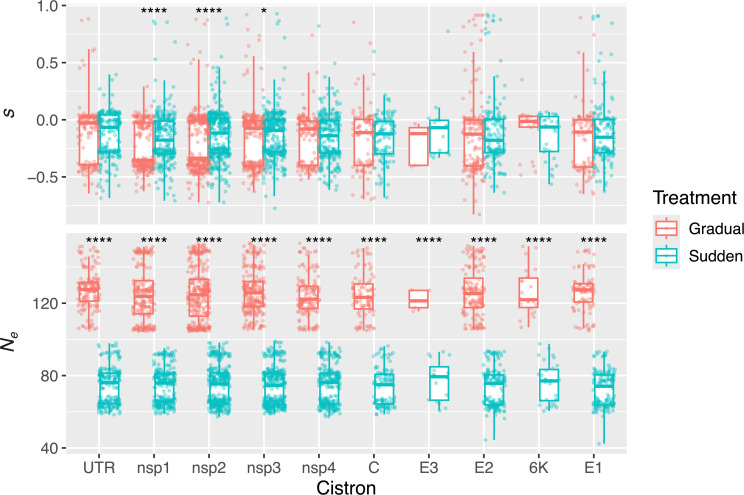
Population parameters *s* and *N_e_* for each allele across all populations. Boxplots of the mean values of the posterior distribution of *s* and *N_e_* for each allele, displayed by treatment and cistron. Asterisks represent the significance of Mann–Whitney two-samples tests: ^*^ *p* < 0.05 and ^****^ *p* < 0.0001.

The selection coefficient (*s*) and effective population size (*N_e_*) were estimated for each allele in the evolving viral populations (figure 2) using a diffusion approximation to Wright–Fisher process [21]. This method is essentially the same approximation developed by Kolmogorov and later analysed by Kimura in the 1940s and 1950 s, as reviewed by Rouzine *et al.* [24]. Regardless of the cistron, the sudden host transition treatment consistently resulted in approximately 2 times smaller *N_e_* values. Because *s* and *N_e_* can influence each other, we tested for the effects of cistron, treatment, population and their interactions on both *s* and *N_e_* parameters. All independent variables and their interactions, with the exception of the interaction between treatment and cistron, were significant (MANOVA; *p* < 0.0001 for treatment, cistron and population, and *p* = 0.0012 for the interaction between cistron and population). By analysing *s* and *N_e_* independently, treatment had the relatively greater effect on *N_e_* but very small effect on *s* ($\eta_{P}^{2}$ *=* 0.99 for *N_e_* and $\eta_{P}^{2}$ = 0.01 for *s*). However, post hoc pairwise tests show that, on average, alleles observed in the sudden transition at the *nsp1*, *nsp2* and *nsp3* cistrons are significantly less deleterious that those found under the gradual transition. The proteins encoded by these three cistrons are involved in the formation of the viral replication complex [25], thus are expected to be under strong purifying selection. In particular, the product of *nsp3* is involved in host specificity and virulence.

Sudden host transitions were associated with stronger (more extreme) bottlenecks, which led to reduced *N_e_* and, in turn, increased the influence of genetic drift. Interestingly, unlike in the gradual treatment, the cistron had no significant effect on *s* (ANOVA; *p* < 0.0001 for the gradual treatment and *p* = 0.3400 for the sudden treatments). One possible explanation is that under strong bottlenecks, the effect of selection across different genomic regions may be weaker or less detectable, whereas it becomes more apparent when the populations are less impacted by drift, as seen in the gradual treatment. However, even in the gradual treatment, the effect size of cistron on *s* was moderate ($\eta_{P}^{2}$ *=* 0.03). Despite significant differences in population parameters between treatments, particularly in *N_e_*, the approxwf method assumes each allele evolves independently and ignores linkage effects such as clonal interference, which have been shown to be important [26]. More sensitive methods for the estimation of population parameters are available [27–29].

### 3.2. A sudden host transition is associated with more instability in population allele composition

**Figure 3.**
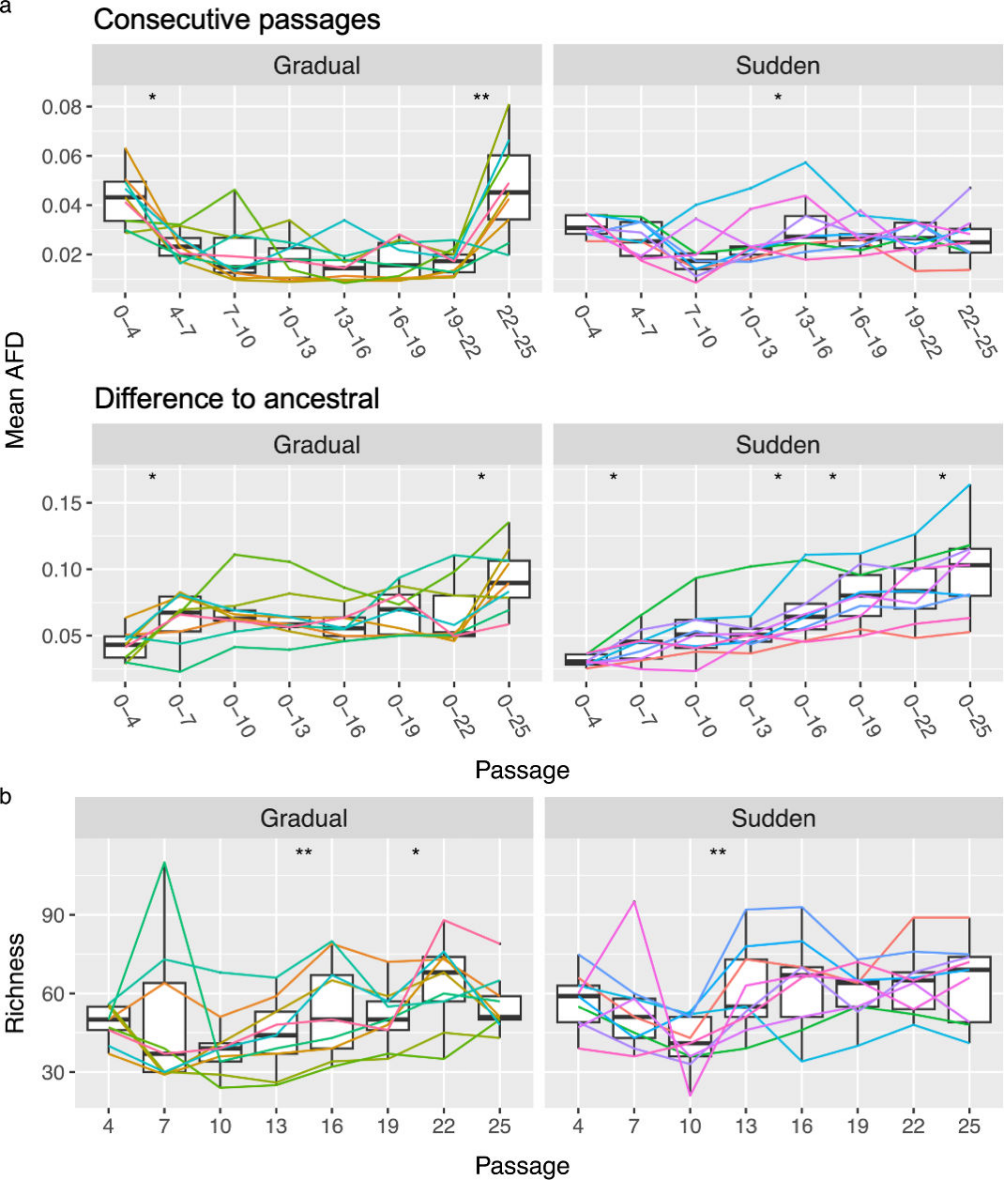
Evolution of the populations’ composition. (a) Boxplots of the mean AFD between two consecutive passages and between each passage and ancestral sequence, also showing the mean AFD for each population represented by lines. (b) Boxplot of the richness of each passage. Lines represent the richness of each population, and asterisks represent the significance of paired samples Wilcoxon tests: ^*^ *p* < 0.05 and ^**^ *p* < 0.01.

Changes in the composition of the populations between consecutive passages were evaluated by summing the AFDs [22] of all alleles. Overall, the composition of the populations was more variable (less steady) in the sudden treatment (figure 3), most likely due to greater effects of genetic drift. In the gradual treatment, most populations changed steadily until the last passage. At this point, a large shift in the composition of the populations was seen (figure 3a).

The rate at which the populations diverged from the ancestral sequence also differed between treatments (figure 3a). In the sudden treatment, populations showed a steady trend of increasing divergence from the ancestor. In contrast, the gradual treatment displayed instances where populations became more similar to the ancestral sequence over time. This phenomenon, known as the leapfrog effect [30], occurs when one haplotype replaces another, with both having evolved from a common ancestor. Since both haplotypes are more similar to their ancestor than they are to one another, the population can become more similar to the ancestral population as it evolves. Five not mutually exclusive mechanisms can be brought forward to explain this leapfrog effect. First, balancing selection that might favour diversity at certain loci, allowing rare haplotypes to gain an advantage under changing environmental or selective conditions [26]. Second, epistasis and hitchhiking in which specific combinations of alleles in rare haplotypes may confer a fitness advantage (epistasis) that becomes beneficial over time, hitchhiking them to dominance [31–33]. Third, environmental fluctuations can alter the fitness landscape, favouring haplotypes that were previously at a disadvantage, even if they are less related to the dominant haplotypes [34–36]. Fourth, genetic drift in small population allows rare haplotypes to randomly increase in frequency, contributing to their rise in dominance over time [37]. And, fifth, lineage sorting, in which low-frequency alleles unrelated to previously dominant ones, but linked through deeper evolutionary events [38], and recombination, which breaks apart haplotypes and creates new advantageous combinations not present in the previously dominant group [39], both contribute to the emergence of novel variants. Since we have observed the leapfrog phenomena in the gradual transition regime (larger *N_e_*; figure 2), we propose that genetic drift may contribute less than the other four mechanisms.

### 3.3. BHK-21 specialists persisted until the complete elimination of BHK-21 cells from the environment

We hypothesize the co-occurrence of BHK-21-specialist, CHO-specialist and generalist haplotypes in the gradual treatment. Clonal interference and competition are expected between and within generalists and specialists, but less so between haplotypes specialized in different host cells. Clonal interference occurs when beneficial mutations are lost due to competition with other beneficial mutations that are present in other haplotypes [30,40,41]. Here, it is more likely that beneficial mutations on generalists will be lost due to competition with specialists, although the opposite may also happen, especially if there is no trade-off in the evolution of generalists [42]. The large shift in population composition seen at the last passage in the gradual treatment suggests that BHK−21-specialists became extinct once BHK-21 cells were completely absent from the environment. We noted, however, that two populations did not seem to follow this trend, at least as strongly, suggesting that in those cases, a large fraction of high-fitness generalists could have emerged (figure 3a).

To better understand the evolution of population composition, we analysed the richness of each sample, defined as the number of polymorphic sites (figure 3b). Specifically, we aimed to determine whether the elimination of BHK-21 specialists was, as expected, associated with a loss in richness. Interestingly, in the sudden treatment, an initial decline in richness was followed by a relatively high and stable level of richness from passage 13 onwards. This observation, along with the concurrent high variability in the population, suggests that many mutations are lost between passages but are continually replenished by new ones. In the gradual treatment, although a decrease in richness was observed at the final point, it is not statistically significant. Therefore, the apparent loss of BHK-21 specialists does not fully account for the substantial shift in population composition observed at the final passage in the gradual treatment.

**Figure 4.**
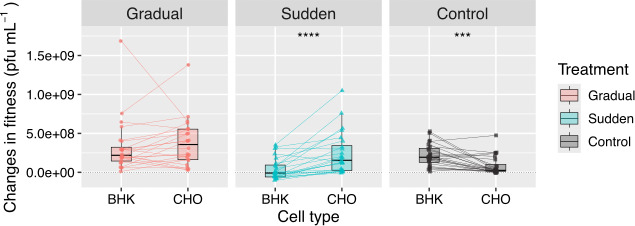
Effect of host replacement regime on fitness. Boxplots of the change in fitness relative to the ancestral for control (lineages evolved in BHK-21 cells), sudden and gradual treatments. Lines represent lineages, and asterisks represent the significance of paired Wilcoxon tests: ^***^ *p* < 0.001 and ^****^ *p* < 0.0001.

We reanalysed fitness data of the evolved populations on BHK-21 and CHO cells to better contextualize the results presented above (figure 4). Populations evolved under the sudden treatment exhibited higher fitness in CHO cells than in BHK-21 cells, consistent with a composition dominated by CHO-specialist haplotypes. In contrast, no significant fitness difference was observed in populations evolved under the gradual replacement regime. Given the absence of a trade-off between hosts in these populations [1], our results suggested that, in the gradual treatment, the combined fitness of CHO specialists and generalists in CHO cells is comparable to the fitness of generalists in BHK-21 cells. Thus, gradual host replacement is associated not only with the emergence of generalist populations but also with the evolution of high-fitness generalist haplotypes [1].

### 3.4. Allele frequencies display large fluctuations and aggregation behaviour

**Figure 5.**
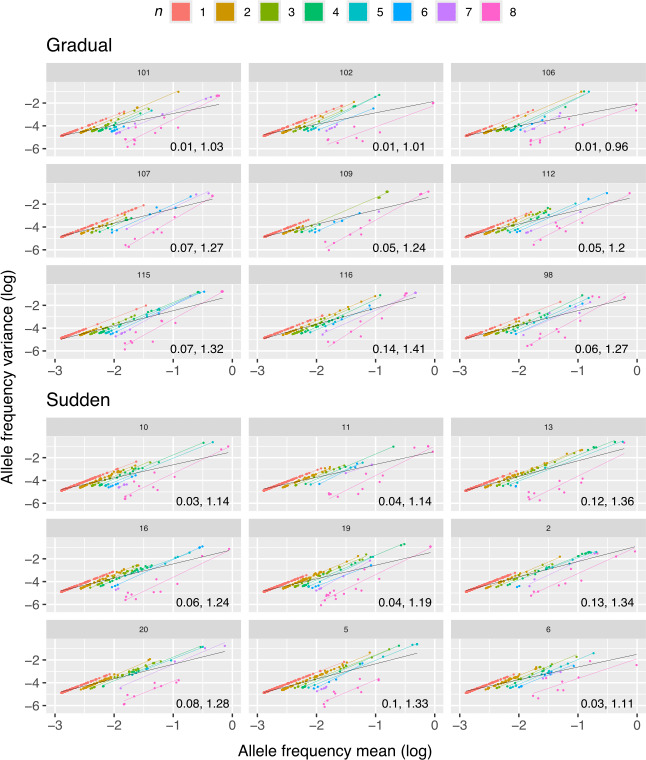
Taylor’s Law plots. Each panel corresponds to one population, where each point corresponds to the mean and variance of an allele frequency across all passages. Alleles are coloured by their number of non-zero occurrences (*n*), and for each *n*, a coloured line represents the fit to Taylor’s Law. A black line represents the fit to Taylor’s Law for all alleles together. Taylor’s parameters *V* and *β*, respectively, for the fit without adjusting for *n*, are shown.

Morley & Turner [2] observed that some positively selected mutations were lost after reaching intermediate allele frequencies, suggesting clonal interference, which was particularly pronounced in the gradual treatment lineages. Here, we investigated whether temporal fluctuations in allele frequencies follow Taylor’s Power Law, focusing on the *β* parameter, which represents the slope of the fit in the log–log space. A *β* value of 1 indicates random fluctuations around the mean, consistent with a Poisson process, while a *β* value of 2 suggests large fluctuations characteristic of an exponential distribution. We hypothesize that due to the larger effect of drift on smaller populations, alleles in the sudden treatment will fluctuate closer to what is expected by the Poisson distribution, and thus, present smaller *β* than alleles in the gradual treatment. Additionally, large fluctuations due to clonal interference, especially for those alleles that reached intermediate or high frequencies, are also expected in the gradual treatment. Inspection of the Taylor’s plots showed that the fraction of zeros had a drastic impact on the fit (figure 5), which is to be expected given that only eight time points are present. Here, it is important to note that the presence of zeros might be either due to biological reasons, i.e. the allele was not present at that time point, or due to technical noise (measurement error). The latter is more likely for low frequency alleles that fluctuate close to the detection limit.

**Figure 6.**
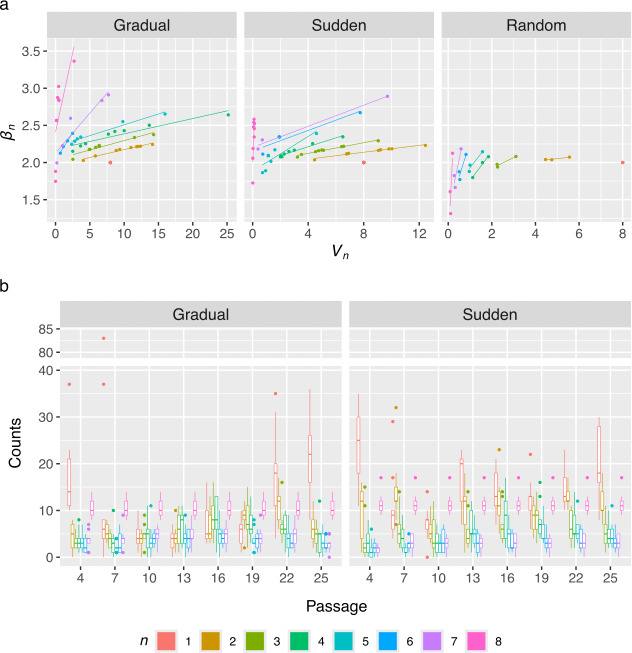
Distribution of alleles frequencies across passages by their non-zero occurrences (*n*). (a) Taylor’s parameters estimated from alleles frequencies for each *n* for the gradual and sudden treatments and random walk matrices. (b) For each passage, boxplots of the distribution of alleles at each category of *n*.

Fits to Taylor’s Law were performed using alleles grouped by their number of non-zero occurrences (*n*) to analyse the scaling of Taylor’s parameters *V* and *β*, referred to here as *V_n_* and *β_n_*, under different host-change regimes. In all cases, *β_n_* was estimated to be close to or higher than one (figure 6a). When fitting based on the number of zeros, we expect that as the mean allele frequency increases, aggregation becomes more apparent, since values are concentrated in non-zero ranges. However, the *β_n_* parameter still effectively captures the distribution of these values and quantifies the degree of aggregation, with an important caveat. Because allele frequency is bounded in the interval (0, 1), early fixation results in high mean frequency and low variance, given that most time points will be ones, causing *β_n_* to decrease and resemble a Poisson-like process. In contrast, late fixation can increase *β_n_*. Therefore, interpretation of this parameter should focus on its deviation from 2, which represents the expected value for random fluctuations without fixation. In other words, mean frequencies and their fluctuations will occupy different regions in respect to *β_n_ = *2 (electronic supplementary material, figure S1a)

To test this, three simulated matrices of random walks were generated based on the cumulative sum of random variables drawn from a normal distribution with mean zero and standard deviations of 0.2, 0.05 and 0.01, respectively (electronic supplementary material, figure S1b), where higher variance corresponds to greater fluctuations between time points. We then performed fits to the simulated random walks and observed that *β_n_* now tends to be lower than one when the frequency of zeros reduces (figure 6a). Additionally, *β_n_* decreases as fluctuation increases, indicating that higher fluctuations are associated to lower *β_n_*. This is due to alleles occupying different regions of the Taylor’s plot depending on the strength of their fluctuations (electronic supplementary material, figure S1c). As expected, when fitting to Taylor’s Law without adjusting for *n*, random matrices behave close to a Poisson distribution (*β* ~ 1), where parameter *V* increases with larger fluctuations.

To better understand how allele frequencies are distributed across the sequenced passages, we examined and plotted the number of alleles separated by *n* at each passage (figure 6b). In the sudden treatment, a higher concentration of alleles with an *n* of one or two was observed during early passages, consistent with a strong bottleneck as viral populations were introduced to the completely new CHO host. Starting at passage 13, the number of these alleles begins to rise again. Overall, the sudden treatment showed a consistently higher number of alleles with an *n* of one or two across all passages compared with the gradual treatment (figure 6b). This pattern aligns with the expected effects of genetic drift, as well as the continuous emergence and loss of new mutations discussed above. In the gradual treatment, alleles with an *n* of one or two also clustered at the first and last passages. However, their early concentration was less pronounced than in the sudden treatment, and their numbers did not begin to rise again until passage 22 (figure 6b).

### 3.5. Effects of treatment and selection coefficient on alleles’ fluctuation

Next, we tested the effect of treatment, population and *n* on the distribution of alleles accounting for all interactions between these variables. The number of non-zero occurrences, *n*, had the highest influence on Taylor’s parameters *V_n_* and *β_n_* (MANOVA; *p* < 0.0001; $\eta_{P}^{2}$ = 0.83). Both the main effect treatment and the interaction between treatment and *n* had a significant influence on Taylor’s parameter (MANOVA; respectively, *p* = 0.0019 and $\eta_{P}^{2}$ = 0.14, and *p* = 0.0080 and $\eta_{P}^{2}$ = 0.11), which indicates that host change rate has a significant and large impact on the distribution of allele frequencies. The main effect population and the interaction between *n* and population were not significant, despite large effect sizes (MANOVA; respectively, *p* = 0.0531 and $\eta_{P}^{2}$ = 0.23, and *p* = 0.0837 and $\eta_{P}^{2}$ = 0.22).

**Figure 7.**
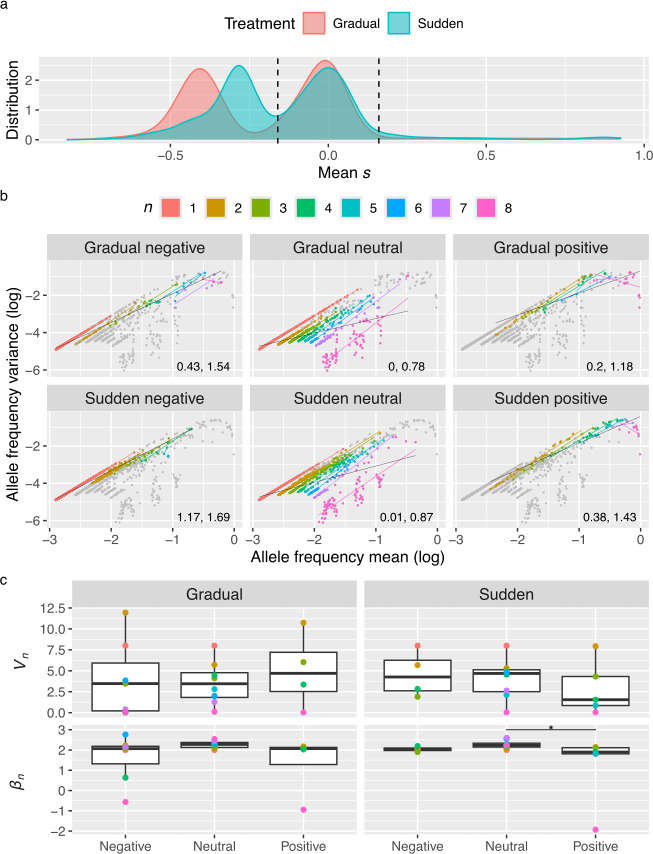
Effects of selection on allele fluctuations. (a) Distribution of *s* per treatment. Dashed lines represent the thresholds for categorizing neutral, negatively and positively selected alleles. Taylor’s parameters *V* and *β*, respectively, for the fit without adjusting for *n*, are shown. (b) Taylor’s plots of combined allele frequencies across populations by treatment and *s*. All the alleles of each treatment are represented as dots and are coloured by their number of non-zero occurrences (*n*) in their selection classification. For each *n* and *s*, a coloured line represents the fit to Taylor’s Law. A black line represents the fit to Taylor’s Law for all alleles classified as the corresponding *s* in the panel. Taylor’s parameters *V* and *β*, respectively, for the fit without adjusting for *n*, are shown. (c) Boxplots of *V_n_* and *β_n_* by treatment and *s* category. Asterisks represent the significance of unpaired Wilcoxon tests: ^*^ *p* < 0.05.

Alleles were divided into three categories, neutral, deleterious and beneficial, to determine whether strength of selection has an impact on Taylor’s parameters. By visualizing the distribution of *s* (figure 7a) it becomes clear that *s* follows a mostly bimodal distribution with a peak for negatively selected alleles and another for neutrally selected ones, with only a fraction being positively selected. Interestingly, negatively selected alleles on the gradual treatment seem to be subjected to stronger negative selection. Based on these distributions, we set a threshold of *s* < −0.16 for alleles to be considered as negatively selected and *s* > 0.16 for them to be considered positively selected, whereas alleles with *s*∈ (−0.16, 0.16) are considered as neutral.

Given that the separation of alleles by *s* drastically reduces sample size in some populations and *n* combinations, fits to Taylor’s Law were performed by treatment, (where frequencies from all population were combined), selection and *n* (figure 7b). Still, sample size drastically affected the fits, especially in cases where alleles with low mean were not present (e.g. positively selected alleles with *n* = 8) to accurately scale how variance changes with the mean. Additionally, some *n* categories are missing entirely for either positively or negatively selected alleles depending on treatment. Again, *n* had the highest influence on Taylor’s parameters *V_n_* and *β_n_* (MANOVA; *p* < 0.0001; $\eta_{P}^{2}$ = 0.75), followed by the interaction between *n* and *s* (*p* = 0.0011; $\eta_{P}^{2}$ = 0.31), and *s* (*p* = 0.0299; $\eta_{P}^{2}$ = 0.2). In this case, treatment did not have a significant impact on Taylor’s parameters. It is likely that combining allele frequencies across populations introduced confounding effects. This is supported by the significant interaction between *n* and population when Taylor’s parameters were estimated separately for each population.

Despite not finding a significant influence of treatment on Taylor’s, some very interesting observations can be made from these Taylor’s plots in regard to *s*. First, points are far more dispersed for neutral alleles, and occupy a region composed by alleles that are present at several time points but have low mean frequency and low variance. Most notably, all alleles with *n* = 8 that have low mean frequency and variance are neutral. Due to this, if the data is fitted to Taylor’s Law without adjusting for *n*, neutral alleles will be closer to a Poisson distribution. Also, neutral alleles exhibit the highest values of *β_n_* (figure 7c). Second, positively selected alleles with an *n* = 8 have negative *β_n_* and their variance decreases as their mean frequency increase. This is due to early fixation, causing most time points to be ones, and consequently, their variance will be low. When fitting the data to Taylor’s Law without adjusting for *n*, this drives *β* down, and as a result, *β* is lower for positively selected alleles in comparison with negatively selected ones. Additionally, positively selected alleles occupy a region of the Taylor’s plot close to the upper limits of the system, especially for higher *n*. Third, negatively selected alleles occupy a region of the Taylor’s plot between neutral and positively selected alleles. This difference is clearer for the sudden treatment.

### 3.6. Host replacement regime affects the distribution of mutations along the Sindbis virus genome

Lastly, it is likely that host change dynamics may also have an effect not only on the temporal aggregation dynamics of allele frequencies but also on genomic location of mutations due to the interplay between drift and selection and linkage. Due to genetic hitchhiking [2,31–33], neutral or low fitness mutations may benefit from being strongly linked to high fitness ones. In addition, the gradual host replacement is associated with greater clonal interference [2], in which genomes containing multiple beneficial mutations outcompete those containing only one, promoting the fixation of linked mutations [2,43–45]. Given that the fixation of sweeps of mutations is greater in the gradual treatment [2], we sought to investigate whether mutations in the gradual treatment are less evenly distributed along the genome, meaning if they are aggregating.

**Figure 8.**
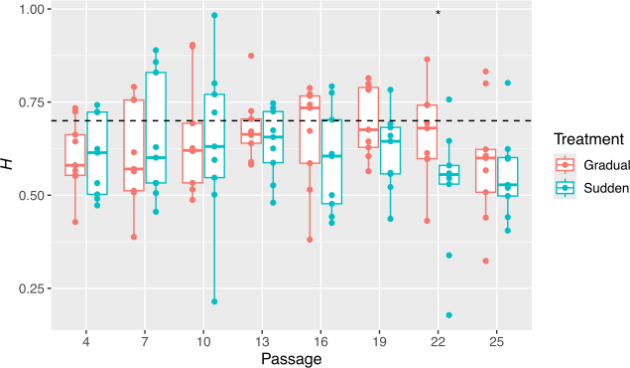
Persistent behaviour in the placement of mutations. Hurst exponents (*H*) estimated from binary sequences (where 1 represent a mutant allele) for each population at each passage. Dashed line represents the 0.7 Hurst phenomenon threshold. Asterisks represent the significance of unpaired Wilcoxon tests: ^*^ *p* < 0.05.

For this, we estimated the empirical Hurst’s exponent *H* to measure persistent behaviour in the placement of mutations at each passage for each population (figure 8). Here, *H* was estimated from binary sequences with the same length as SINV genome, where ones represent sites with any disagreement to the ancestral sequence. Lower values of *H* are associated with more evenly spaced mutations along the SINV genome. Despite high variability in the estimates, particularly in the sudden treatment, evidence of persistent behaviour was observed in most cases, with the median *H* value at each passage remaining above 0.5 for both treatments (figure 8). During the first two passages, the median *H* was higher in the sudden treatment, followed by two passages where *H* values were similar between treatments. In the final passages, *H* decreased and became lower in the sudden treatment; however, these differences were only statistically significant at passage 22 (*p* = 0.040). This pattern suggests that the continuous emergence and loss of mutations observed in the sudden treatment at later passages is associated with a more random distribution of mutations along the genome, potentially explaining the more scattered mutation pattern in this treatment. Nonetheless, treatment did not have a significant overall effect on *H* (linear model with passage and treatment as orthogonal fixed effects, and population as a random effect nested within treatment; sudden coefficient estimate = −0.043, *p* = 0.100).

## 4. Conclusions

This study investigates how viruses adapt at the molecular level when exposed to novel hosts. By reanalysing genomic data from an experimental evolution study of SINV under sudden versus gradual host-replacement regimes, we explored how environmental heterogeneity, genetic drift and selection shape temporal allele frequency fluctuations and the genome-wide distribution of mutations. Using two tools from the complex systems framework, Taylor’s Power Law and the Hurst exponent, we examined whether the observed fluctuations reflect random noise or more complex underlying dynamics.

Our findings highlight the pivotal role of environmental heterogeneity in viral adaptation. Gradual host replacement fosters more stable and convergent evolutionary trajectories, enhancing viral fitness across hosts. This regime promotes clonal interference, facilitating the fixation of linked beneficial mutations and producing temporally persistent mutation patterns across the genome. In contrast, sudden host transitions reduce effective population size, intensify genetic drift, destabilize allele frequencies and result in more dispersed mutation distributions along the viral genome. These results underscore how both the rate and nature of environmental change influence evolutionary outcomes.

The application of Taylor’s Power Law and the Hurst exponent provided insights into the scaling behaviour and temporal aggregation of allele frequency fluctuations. Neutral alleles exhibited higher variability, while beneficial alleles tended to reach fixation earlier. The interplay between genetic drift, selection and linkage effects shaped the spatial and temporal distributions of mutations. Gradual host replacement favoured aggregated mutation patterns due to hitchhiking of linked beneficial mutations. While our findings provide valuable insights into the evolutionary dynamics of SINV under different host-replacement regimes, we acknowledge that the relatively small sample sizes, particularly the limited number of replicate populations and the eight sequenced time points, may constrain the generalizability and statistical power of some analyses. This limitation is especially relevant when estimating Taylor’s parameters and Hurst exponents, or when stratifying alleles by selection category and occurrence frequency. As such, some subgroup trends should be interpreted with caution. Future studies incorporating denser temporal sampling and larger replicate numbers would enhance the robustness of these scaling analyses and allow for more nuanced exploration of stochastic versus deterministic forces in viral evolution.

Finally, while gradual host-replacement led to greater convergence on high-fitness generalist haplotypes, the more erratic evolutionary trajectories seen under sudden host transitions may, over the long term, facilitate the emergence of high-fitness haplotypes that are distant from local optima in sequence space. This may result from the persistence of neutral or mildly deleterious mutations and the broader genomic spread of mutations under this regime.

Overall, this study advances our understanding of virus evolution and offers a novel framework for predicting pathogen adaptation to dynamic environments, such as shifts in host availability or immune pressures. However, we recognize that these findings may not generalize across all viral systems. In natural populations, particularly those involving multicellular hosts, bottlenecks during host-to-host transmission, immune responses, and spatial structure can have pronounced effects on allele dynamics. Additionally, intrinsic factors such as mutation rates, recombination frequency and the number of accessible beneficial mutations vary widely across viruses and are likely to influence adaptive trajectories. While the principles uncovered here, such as the impact of environmental change rate on effective population size and allele frequency fluctuations, may extend to other RNA viruses, further studies across diverse viral systems and ecological contexts are needed to test the universality of these patterns. Finally, we acknowledge that the relatively small number of replicate populations and limited temporal resolution may constrain the statistical power of some analyses, particularly those involving Taylor’s parameters and Hurst exponents. Future work with denser sampling and larger experimental designs will be essential to validate and expand upon these findings.

## Data Availability

Raw sequencing data are available through the NCBI SRA project accession number SRP096731. Electronic supplementary material are available at the Zenodo repository [46].
